# Supplementary material for: Transmembrane chemokines act as receptors in a novel mechanism termed inverse signaling
Source: eLife. 2016 Jan 21;5:e10820. doi: 10.7554/eLife.10820 (PMC4739769; doi:10.7554/eLife.10820)
Supplement: Figure 3—source data 1. — DOI: http://dx.doi.org/10.7554/eLife.10820.008 [file elife-10820-fig3-data1.docx]

**Figure 3 – source data 1**

| **U343** | |  | |  | |  | |  | |
| --- | --- | --- | --- | --- | --- | --- | --- | --- | --- |
|  | | colonized area = [scratch area 0h]-[scratch area 24 h] | | | | | | | |
|  | | Control | | CXCL16 | | 20% FCS | |  | |
| Mean Exp 1 | | 332.20 | | 350.40 | | 836.60 | |  | |
| Mean Exp 2 | | 212.40 | | 233.20 | | 698.98 | |  | |
| Mean Exp 3 | | 265.25 | | 242.00 | |  | |  | |
|  | |  | |  | |  | |  | |
| Total Mean | | 269.95 | | 275.20 | | 767.79 | |  | |
| SD | | 60.04 | | 65.27 | | 97.31 | |  | |
|  | |  | |  | |  | |  | |
| Mean % | | 100.0% | | 101.9% | | 284.4% | |  | |
| Mean SD | | 22.2% | | 24.2% | | 36.0% | |  | |
|  | |  | |  | |  | |  | |
| **A764** | |  | |  | |  | |  | |
|  | | colonized area = [scratch area 0h]-[scratch area 24 h] | | | | | | | |
|  | | Control | | CX3CL1 | | 20% FCS | |  | |
| Mean Exp 1 | | 260.83 | | 271.62 | | 581.31 | |  | |
| Mean Exp 2 | | 303.29 | | 307.26 | | 565.76 | |  | |
| Mean Exp 3 | | 261.32 | | 210.08 | |  | |  | |
|  | |  | |  | |  | |  | |
| Total Mean | | 275.15 | | 262.99 | | 573.54 | |  | |
| SD | | 24.37 | | 49.16 | | 11.00 | |  | |
|  | |  | |  | |  | |  | |
| Mean % | | 100.0% | | 95.6% | | 208.4% | |  | |
| Mean SD | | 8.9% | | 17.9% | | 4.0% | |  | |
|  | |  | |  | |  | |  | |
|  |  | |  | |  | |  | |  |
